# Changes in Expression of Aquaporin-4 and Aquaporin-9 in Optic Nerve after Crushing in Rats

**DOI:** 10.1371/journal.pone.0114694

**Published:** 2014-12-05

**Authors:** Hiroyuki Suzuki, Hidehiro Oku, Taeko Horie, Seita Morishita, Masahiro Tonari, Kazuma Oku, Akiko Okubo, Teruyo Kida, Masashi Mimura, Masanori Fukumoto, Shota Kojima, Shinji Takai, Tsunehiko Ikeda

**Affiliations:** Department of Ophthalmology, Osaka Medical College, Osaka, Japan; Instituto Murciano de Investigación Biosanitaria-Virgen de la Arrixaca, Spain

## Abstract

The purpose of this study was to determine the temporal and spatial changes in the expression of AQP4 and AQP9 in the optic nerve after it is crushed. The left optic nerves of rats were either crushed (crushed group) or sham operated (sham group), and they were excised before, and at 1, 2, 4, 7, and 14 days later. Four optic nerves were pooled for each time point in both groups. The expression of AQP4 and AQP9 was determined by western blot analyses. Immunohistochemistry was used to determine the spatial expression of AQP4, AQP9, and GFAP in the optic nerve. Optic nerve edema was determined by measuring the water content in the optic nerve. The barrier function of the optic nerve vessels was determined by the extravasated Evans blue dye on days 7 and 14. The results showed that the expression of AQP4 was increased on day 1 but the level was significantly lower than that in the sham group on days 4 and 7 (*P*<0.05). In contrast, the expression of AQP9 gradually increased, and the level was significantly higher than that in the sham group on days 7 and 14 (*P*<0.05, Tukey-Kramer). The down-regulation of AQP4 was associated with crush-induced optic nerve edema, and the water content of the nerve was significantly increased by 4.3% in the crushed optic nerve from that of the untouched fellow nerve on day 7. The expression of AQP4 and GFAP was reduced at the crushed site where AQP4-negative and AQP9-positive astrocytes were present. The barrier function was impaired at the crushed site on days 7 and 14, restrictedly where AQP4-negative and AQP9-positive astrocytes were present. The presence of AQP9-positive astrocytes at the crushed site may counteract the metabolic damage but this change did not fully compensate for the barrier function defect.

## Introduction

The aquaporins (AQPs) are hydrophobic membrane proteins that permit water to be transported across cell membranes. Various aquaporins are present in the central nervous system (CNS) including the retina and optic nerve [1], [2]. AQP4, the main water channel protein in the CNS, is chiefly expressed on the end-feet of astrocytes which envelope the capillaries in the brain [3]. AQP4 plays an important role in maintaining water and ion homeostasis which is altered by neuronal activity [4]. For example, inwardly rectifying K^+^ (Kir) channels coexist with AQP4 on the end-feet of astrocytes, and they remove extracellular potassium ions (K^+^) generated by neuronal activity [5]. Water flux through the AQP4 channels is linked to this K^+^ spatial buffering, and thus AQP4 can control the extracellular water and ion homeostasis. Along with astrocytes, Müller cells in the retina also play important roles in maintaining the extracellular water and ion homeostasis through AQP4 [6], [7]. In addition, AQP4 is important for maintaining the integrity of the blood-brain barrier [8].

AQP9 is highly permeable to water and various solutes including lactate [9]. AQP9 may function in maintaining the energy supply to optic nerve axons in the form of lactate [10]. In addition, AQP9 is the only water channel that is expressed at the optic nerve head [11]. Interestingly, AQP9 is up-regulated in the ischemic brain [12], which would enhance lactate clearing with water flux from the extracellular space under pathological conditions.

The AQPs are closely associated with edematous changes in the CNS. Removal of AQP4 decreases the brain edema caused by acute water intoxication [13], and it is essential for water to be removed continuously during intraparenchymal fluid infusion [14]. These characteristics suggest that AQP4 contributes to both the formation and resolution of brain edema.

The optic nerve becomes edematous under various conditions including traumatic injury and inflammation. Because the optic nerve is enveloped within the optic nerve sheath and passes through the optic canal, there is very limited space for swelling. Thus, optic nerve edema can cause axonal damage, and changes in the level of AQPs in the optic nerve following various injuries need to be determined. Changes of AQP4 and AQP9 in the retina have been demonstrated under diabetic and ischemic conditions [15]–[22]. Changes of AQP9 were also found in the optic nerve head after an increase in the intraocular pressure [11], indicating that they are probably involved in the pathogenesis of diabetic retinopathy, retinal ischemia, and glaucoma. However, how the levels of AQPs are changed after optic nerve injury has not been determined. The results of earlier studies are contradictory as to whether AQP4 is up-regulated [23] or down-regulated [24] after the optic nerve is crushed. In addition, the AQPs may play a role in the induction of reactive astrogliosis in response to optic nerve damage because the AQPs are known to be associated with migration and proliferation of astrocytes [25].

These findings suggested that both AQP4 and AQP9 play important roles in the repair of the optic nerve after injury, but their temporal and spatial expression patterns are different. These changes need to be determined to understand the mechanism causing the edema after traumatic optic nerve injury. In addition, determining the mechanisms of these changes may lead to new therapeutic interventions.

Thus, we hypothesized that changes in the AQP4 levels are associated with the development of optic nerve edema, and AQP9 will change in a manner which reflects a compensatory mechanism for the metabolic damage. To test this hypothesis, we determined the temporal and spatial changes of AQP4 and AQP9 expressions after the optic nerve is crushed in rats. Rats were killed on days 1, 2, 4, 7, and 14 after crushing the optic nerve and the optic nerves were collected. The levels of AQP4, AQP9 and GFAP were determined by western blot analyses, and immunohistological analyses were performed to determine localization of these proteins around the crushed sites. Optic nerve edema was serially determined by the water content of the optic nerves on days 1, 4, 7, and 14. In addition, the blood-optic nerve barrier function was determined by the extravasation of Evans blue dye injected into the femoral artery on days 7 and 14.

## Materials and Methods

### Animals

Nine-week old, male Wistar rats weighing between 180–200 g were purchased from Japan SLC (Shizuoka, Japan), and housed in an air-conditioned room with a temperature of approximately 23°C humidity of 60%. The room lights were on a 12∶12 light∶dark cycle. The animals had free access to food and water, and they were handled in accordance with the ARVO Resolution for the Use of Animals in Ophthalmic and Vision Research. The experimental protocol, which conformed to the Animal Research: Reporting *In Vivo* Experiments (ARRIVE) guidelines [26], was approved by the Committee of Animal Use and Care of the Osaka Medical College (No. 25055). A total of 87 rats were used, and all rats survived until the completion of the scheduled protocol.

### Anesthesia and euthanasia

All surgeries were performed under general anesthesia by intraperitoneal injection of pentobarbital sodium (50 mg/kg body wt), and all efforts were made to minimize suffering. Rats were euthanized by exposure to CO_2_ at a rate of 6 L/min in a cage (13.8 L) with wood-shaving bedding. In some cases, rats were euthanized by cardiac perfusion under deep general anesthesia by intraperitoneal pentobarbital sodium (150 mg/kg body wt).

### Chemicals

Unless noted, chemicals were purchased from Sigma-Aldrich (St. Louis, MO, USA).

### Optic nerve crushing

Animals were anesthetized with an intraperitoneal injection of pentobarbital sodium (50 mg/kg). A skin incision was made along the midline of the skull to expose the superior surface of the left eye. The superior rectus muscle was incised to expose the left optic nerve, and the left optic nerve was crushed with forceps 2 mm behind the eye for 10 seconds [27]. Care was taken not to occlude the blood vessels and cause retinal ischemia. We confirmed by indirect ophthalmoscopy that the retinal circulation was not blocked and also verified by real-time PCR that the HIF-1α gene was not up-regulated [28]. A sham operation was performed on the left eyes of other animals by exposing the optic nerve in the same way but not crushing the optic nerve. The right eyes were untouched in both treatments.

### Determination of optic nerve edema

The degree of optic nerve edema after the optic nerve was crushed was determined by measuring the water content in the optic nerve. The left optic nerves were crushed under general anesthesia by an intraperitoneal injection of 50 mg/kg of pentobarbital sodium, and the right optic nerves were untouched. The rats were euthanized by CO_2_ inhalation on days 1, 4, 7, and 14 after the crushing (n = 4 at each time point). The optic nerves (approximately 4 mm) were excised from both eyes. The wet weight of the specimen was measured immediately after the excision, and the dry weight was measured after drying the optic nerves in a sterilization oven at 60°C for 96 hrs. The water content was determined by subtracting the dry weight from the wet weight, and the water content was expressed as the percentage to the wet weight. The water content of the crushed optic nerves was compared with that of the untouched fellow nerves. A total of 16 rats were used for this experiment.

### Immunoblotting

Immunoblotting was used to determine the temporal changes in the expressions of AQP4, AQP9, and GFAP in the crushed optic nerve. Animals were divided into crushed and sham groups. They were euthanized by CO_2_ inhalation on days 1, 2, 4, 7, and 14 after crushing the optic nerves (n = 4 for each time point for both groups). Approximately 4 mm of the optic nerve centered on the crushed site was excised. The 4 optic nerves were pooled as one sample at each time point, and they were homogenized in lysis buffer containing 1.0 mM phenylmethanesulfonyl fluoride, 10 µM pepstatin A,10 µM leupeptin,10 µM aprotinin, 0.1% SDS, 1.0% Nonidet P-40, 5.0% sodium deoxy cholate, 50 mM Tris-HCl (pH 7.6), and 150 mM NaCl. The suspension was centrifuged, and the supernatant was used to determine the total protein concentration by the Lowry method (DC Protein Assay Reagent, Bio-Rad, Hercules, CA, USA).

Samples were separated on a 15% SDS-polyacrylamide gel and transblotted onto PVDF membranes. The membranes were blocked with 5% skim milk in TBS-T (pH 7.4, 0.1% Tween 20) followed by overnight incubation with a rabbit polyclonal anti-AQP4 (sc-20812), mouse monoclonal anti-AQP9 (sc-74409, Santa Cruz, Dallas, USA), or rabbit monoclonal anti-GFAP (EP672T, 1∶500, Merck Millipore, Billerica, MA, USA) antibody at 4°C. Tubulin (α-tubulin, 1∶1000; Merck Millipore) was used as an internal control. The protein bands were made visible by horseradish peroxidase-conjugated goat anti-rabbit or goat anti-mouse IgG (1∶2000, Promega, Madison, WI, USA). The signals were intensified with an ECL plus Western blotting detection system (GE Healthcare, Amersham, UK). The densities of the bands of proteins were quantified with a luminescent image analyzer (LAS-3000, Fujifilm, Tokyo, Japan). The levels of expression of these protein were quantified with the embedded software (Multi Gauge version 3.0) and standardized to the baseline level. The assays were run in triplicate at each time point using the pooled samples. A total of 48 rats were used for the western blot analyses.

### Immunohistochemistry

On days 1, 4, 7, and 14 post-crushing, rats were deeply anesthetized with an intraperitoneal injection of pentobarbital sodium (150 mg/kg) and perfused through the left ventricle of heart with saline (200 ml) followed by 4% paraformaldehyde (PFA) in 0.1 M phosphate buffer at pH 7.4 (300 ml) using a Masterflex pump (Cole-Parmer 7530-70, Chicago, IL, USA). After removing the skull and cerebral hemispheres, the optic nerves were carefully removed and post-fixed in 4% PFA in PBS overnight. After washing with PBS, the tissues were immersed in 30% sucrose overnight at 4°C and then embedded in OCT compound (BDH Laboratory Supplies, Poole, UK). Then, 10 µm frozen sections were cut with a cryostat. After blocking the sections with 1.0% normal goat or donkey serum plus 1.0% BSA and 0.1% triton-X 100 in PBS, the sections of the optic nerves were incubated with primary antibodies of rabbit polyclonal anti-AQP4 (sc-20812, 1∶500), mouse monoclonal anti-AQP9 (sc-74409, 1∶500, Santa Cruz), rabbit polyclonal anti-GFAP (AB5804, 1∶500, Merck Millipore), or mouse monoclonal anti-GFAP antibody (G3893, 1∶500, Sigma) overnight at 4°C. In some experiments, the optic nerves were exposed to mouse monoclonal anti-CD68 antibody (1∶500, Serotec, Oxford, UK), rabbit polyclonal anti-CD68 antibody (ab-125212, 1∶500, Abcam, Cambridge, MA, USA) or goat polyclonal anti-nestin (sc-21249, 1∶200, Santa Cruz). These sections were incubated for 2 hrs at room temperature in Alexa 594 or Alexa 488-conjugated to the appropriate secondary antibodies (Invitrogen, Carlsbad, CA, USA) diluted by 1∶500.

Fifteen rats were used for immunohistochemistry with 3 rats at 1, 4, 7 and 14 days after crushing the optic nerve plus 3 control rats.

### Determination of blood-optic nerve barrier function

On days 7 and 14 after crushing the optic nerves or sham operation, rats (n = 2 each for crushing and sham operation on days 7 and 14) were anesthetized with an intraperitoneal injection of pentobarbital sodium (50 mg/kg). They were injected in the femoral artery with 2% Evans blue (5.0 ml). Thirty minutes were allowed for the dye to leak from the injured vessels into the optic nerve tissue. After additional intraperitoneal injection of pentobarbital sodium (100 mg/kg), formalin was perfused into the heart for fixation, and optic nerves were excised and sectioned as described. The extravasated Evans blue-albumin complex yielded a red fluorescence in unstained optic nerve sections. A total of 8 rats were used for the evaluation of the blood-optic nerve barrier function. The processed sections were examined and photographed with a fluorescent microscope (BZ 8000, Keyence, Osaka, Japan).

### Statistical Analyses

The data are expressed as the means ± standard deviations (SDs). Statistical analysis was performed by one-way analysis of variance (ANOVA), and if significant changes were detected, the Tukey-Kramer post-hoc test was done for statistical comparisons among groups. The level of significance was set at *P*<0.05.

## Results

### Changes in expression of AQP4, AQP9, and GFAP after optic nerve crushing

**Figure 1:**
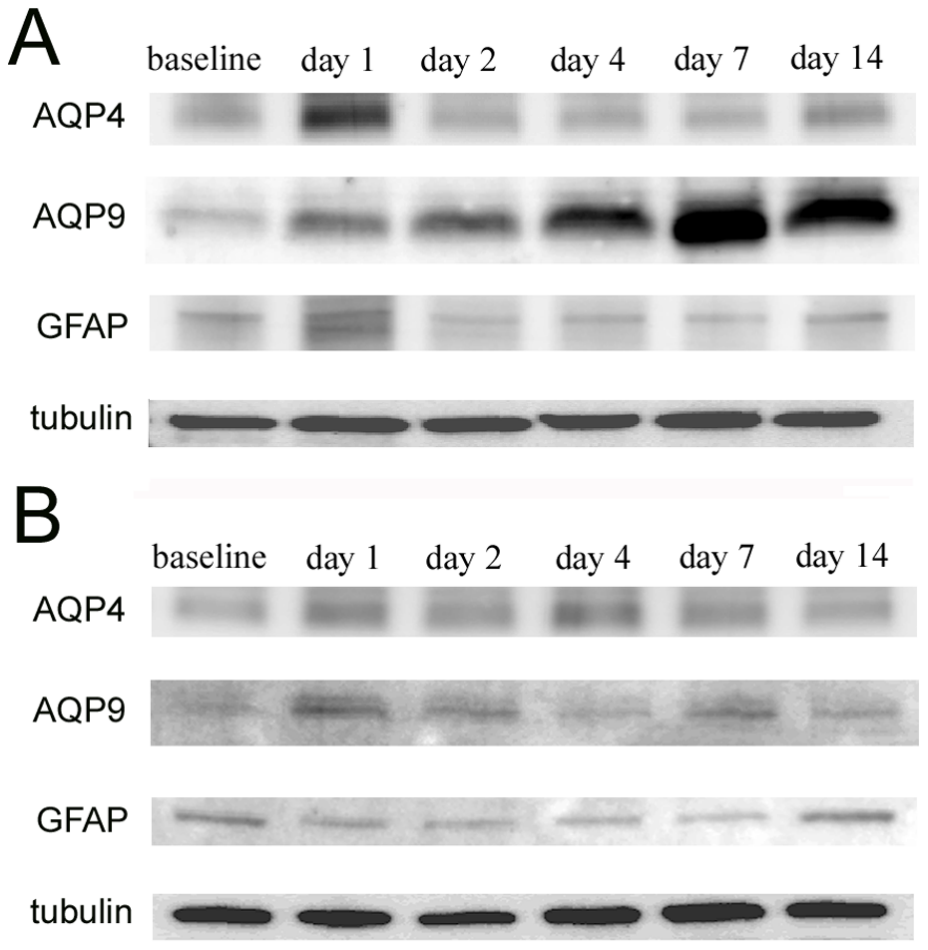
Representative western blots for aquaporin-4 (AQP4), aquaporin-9 (AQP9), and GFAP in extracts from the optic nerves after they were crushed (A) and sham (B) operated. Tubulin was used as an internal control.

**Figure 2:**
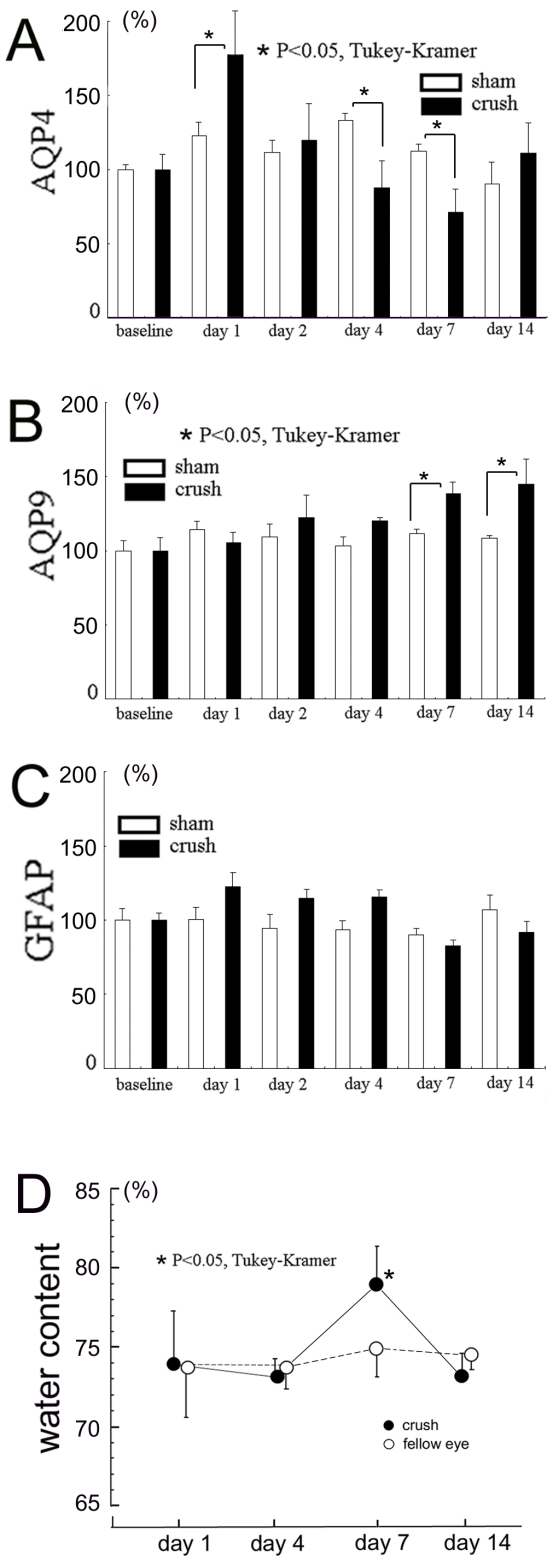
Western blot analyses for AQP4 (A), AQP9 (B), and GFAP (C). A.-C. Changes in the protein levels of AQP4 (A), AQP9 (B), and GFAP (C). Four optic nerves (4 mm in length) centered on the crush sites were serially excised and pooled as one sample at each time point. Proteins were extracted from the pooled samples and assays were run in triplicate at each time point using the pooled samples. D. Changes of water content in the optic nerve. Water content is expressed as percentages to the wet weight (n = 4 at each time point). Data are the means ± standard deviations (SDs). Asterisks indicate significant differences between the crushed optic nerve and the untouched fellow nerve at each time point (*P*<0.05, ANOVA followed by Tukey-Kramer).

The temporal changes of AQPs and GFAP in the optic nerve after crushing were determined by western blot analyses. Western blot analyses of the extracts from the optic nerves of rats that had their optic nerves crushed (A) or sham operated (B) are shown in Figure 1. The levels in the untreated animals in both groups were set as the baseline levels. The levels of AQPs and GFAP were standardized by the α-tubulin expression, and the levels relative to the baseline are shown in Figure 2. For each assessment, there were 6 time points including the baseline in both the crushed and sham groups, for a total of 12 subgroups. The differences among these subgroups were analyzed by one-way ANOVA followed by Tukey-Kramer test.

The level of AQP4 protein increased to 179.3±25.3% of the baseline on day 1 after the optic nerve was crushed (Figure 1A). However, the levels decreased to 87.5±15.3% and 71.6±12.8% of the baseline on days 4 and 7, respectively. Then the levels recovered to the baseline levels on day 14. The AQP4 levels also changed in the sham group but the changes were smaller than that in the crushed group. Specifically, the AQP4 expression was increased to 122.8±9.0%, 133.0±5.1%, and 112.4±4.7% from the baseline on days 1, 4, and 7 respectively in the sham group (Figure 1B). The differences in the AQP4 levels among the subgroups were significant (*P*<0.001, ANOVA) after the optic nerve was crushed (Figure 2A). When the crush and sham groups were compared at each time point, the level of AQP4 was significantly (*P*<0.05) higher in the crushed group than in the sham operated group by 56.5% on day 1, while the level was significantly lower (*P*<0.05, Tukey-Kramer) in the crushed group than in the sham operated group by 45.5% and 40.8% on days 4 and 7, respectively (Figure 2A). The difference was no longer significant on day 14.

In contrast, the level of AQP9 increased slowly after the optic nerve was crushed with a peak on day 14, when the level was 144.9±19.9% of the baseline (Figure 1A). During the experimental period, the levels of AQP9 were stable in the sham group (Figure 1B). The differences in the AQP9 levels among the subgroups assessed at different time points were also significant (*P*<0.001, ANOVA; Fig. 2B). The level of AQP9 was significantly higher in the crushed group than in the sham operated group on days 7 and 14 (*P*<0.05, Tukey-Kramer; Figure 2B).

The changes in the level of GFAP were similar to the changes of AQP4 in the crushed group, i.e., increased on day 1 and decreased on day 7 (Figures 1 and 2C). However, the GFAP levels in the crushed group were not significantly different from that in the sham control group at each time point (*P*>0.05, Tukey-Kramer, Figure 2C).

### Optic nerve edema

The water content in the optic nerve after crushing is shown in Figure 2D. The differences in the water content of the crushed optic nerves from that of untouched fellow nerves were considered to be due to crush-induced optic nerve edema. The levels were similar between the crushed and the untouched fellow nerves on days 1 and 4. There was a significant (*P*<0.05, Tukey-Kramer) increase (4.3%) of water content in the crushed optic nerve (79.3±2.4%) from that of the untouched fellow nerves (75.0±1.8%) on day 7 but the increase was not significant on day 14. Thus, the crush-induced edema was observed only on day 7.

### Immunohistochemistry

**Figure 3:**
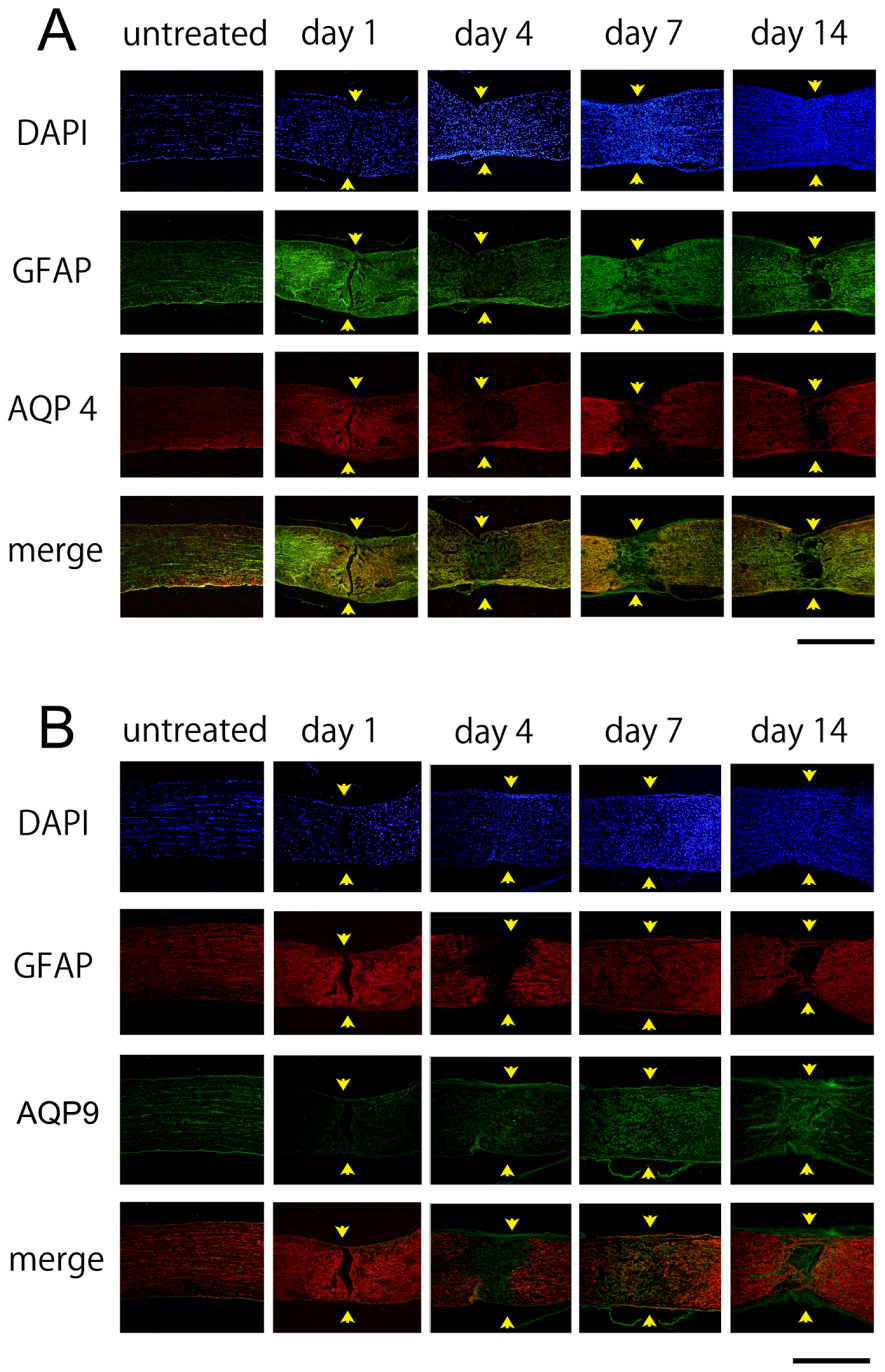
Immunohistochemistry for AQP4 (A) and AQP9 (B) expression before, and 1, 4, 7, and 14 days after crushing the optic nerves. Representative photographs from 3 independent samples are presented. Arrows indicate crush sites. A. Immunoreactivity to AQP4 is increased at the cracks (arrows) caused by the crushing on day 1. Immunoreactivities to AQP4 and GFAP are reduced at the crush sites (arrows) on day 4 and thereafter. Bar  = 500 µm. B. Immunoreactivity to AQP9 is faint along the crush-induced cracks (arrows) on day 1. However, immunoreactivity to AQP9 is intensified at the crush sites on day 4 and thereafter. Bar  = 500 µm.

**Figure 4:**
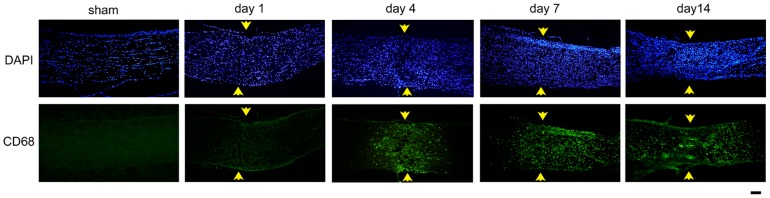
Immunohistochemistry for CD68 in the optic nerve. There is a cellular accumulation indicated by DAPI at the crushed sites of the optic nerve. Some of the cells are positively stained with CD68 suggesting that there is a recruitment of microglia/macrophages to the crushed sites. Bar  = 100 µm.

Representative photomicrographs of the changes in the expression of AQP4, AQP9, and GFAP after crushing the optic nerves are shown in Figure 3. Changes in the expression of CD68, a constitutive maker of microglia/macrophages are shown in Figure 4. Immunoreactivity to AQP4 was intensified along the crush-induced cracks on day 1 (Figure 3A), however cellular infiltration was not detected on day 1 (Figure 4). There was an accumulation of CD68 cells at the crushed site on days 4, 7, and 14 (Figure 4). Along with these changes, the immunoreactivity to GFAP was decreased on day 4 at the crushed site. The degree of immunoreactivity to AQP4 changed in a similar manner as that of GFAP. Thus, the immunoreactivity to AQP4 and GFAP became weaker at the crushed site than at the areas surrounding the lesion on days 4, 7, and 14 (Figure 3A). On the other hand, the immunoreactivity to AQP9 increased at the crushed site where immunoreactivity to GFAP was weak on day 4 and thereafter (Figure 3B).

**Figure 5:**
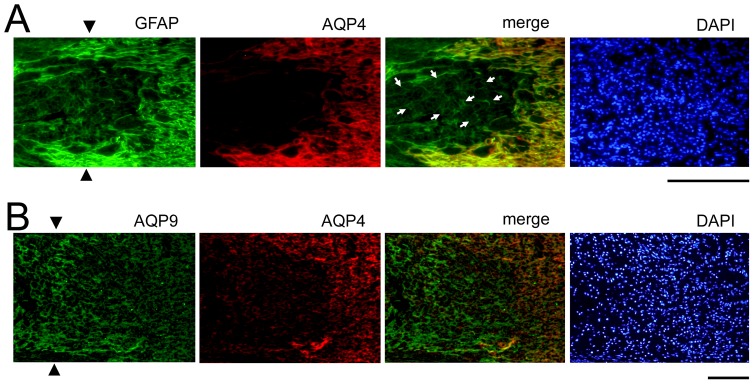
Immunohistochemistry for AQP4, AQP9, and GFAP at the crushed site on day 7. Representative photographs from 3 independent samples are presented. A. Some GFAP-positive cells still exist at the crushed site (arrowheads), where immunoreactivity to AQP4 is almost completely lost indicating the presence of AQP4-negative astrocytes (arrows) at the crushed site. Bar  = 100 µm. B. Immunoreactivity to AQP9 is intensified at the crushed site (arrowheads) where immunoreactivity to AQP4 is clearly reduced. Bar  = 100 µm.

Double labeling with AQP4 and GFAP showed that the AQP4-negative astrocytes were present at the crush site on day 7 (Figure 5A). Double labeling of AQP4 and AQP9 showed that the expression of AQP9 was intensified at the crushed site where the expression of AQP4 was decreased (Figure 5B).

**Figure 6:**
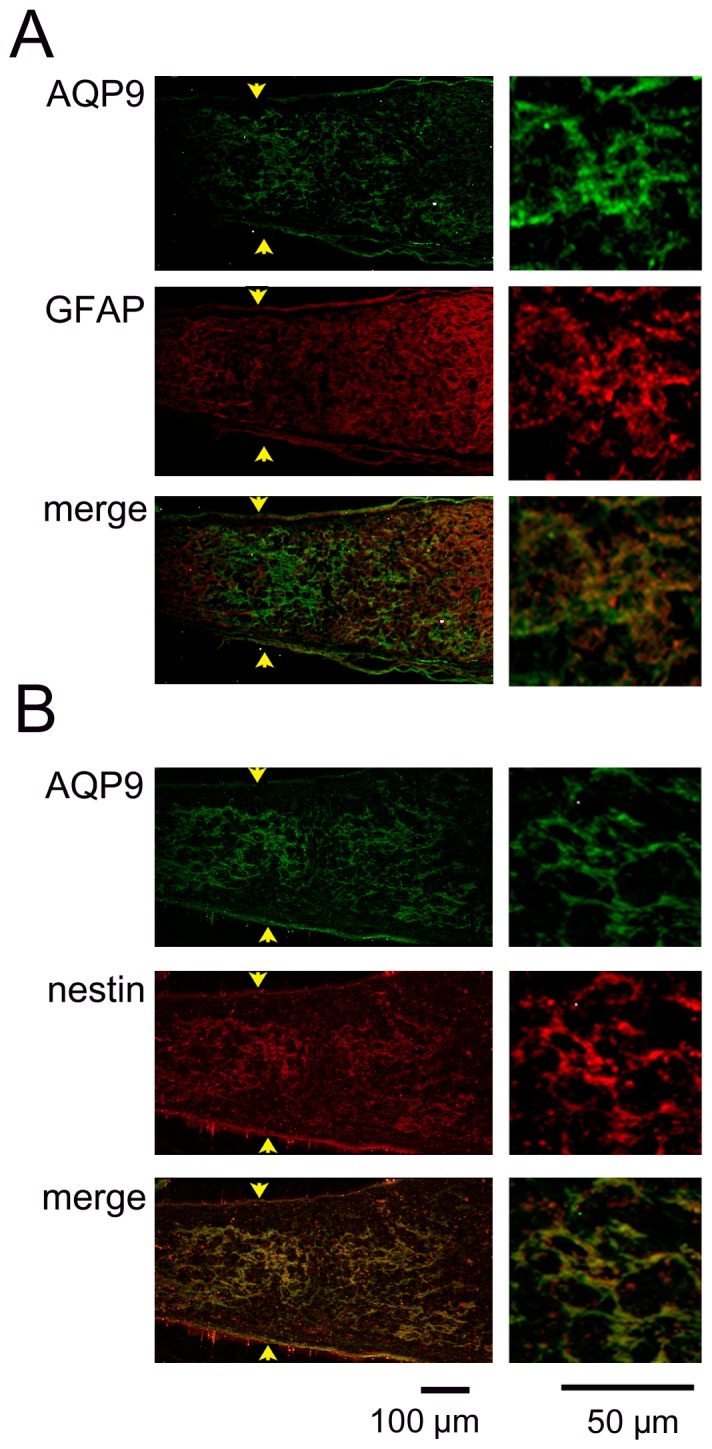
Immunohistochemistry for AQP9, GFAP, and nestin at the crushed site on day 7. Representative photographs from 3 independent samples are presented with higher magnifications in the right column. A: Immunoreactivity to AQP9 has a honeycomb appearance and is co-localized in the GFAP-immunopositive regions. B: Immunoreactivity to AQP9 is also co-localized with immunopositive regions to nestin.

**Figure 7:**
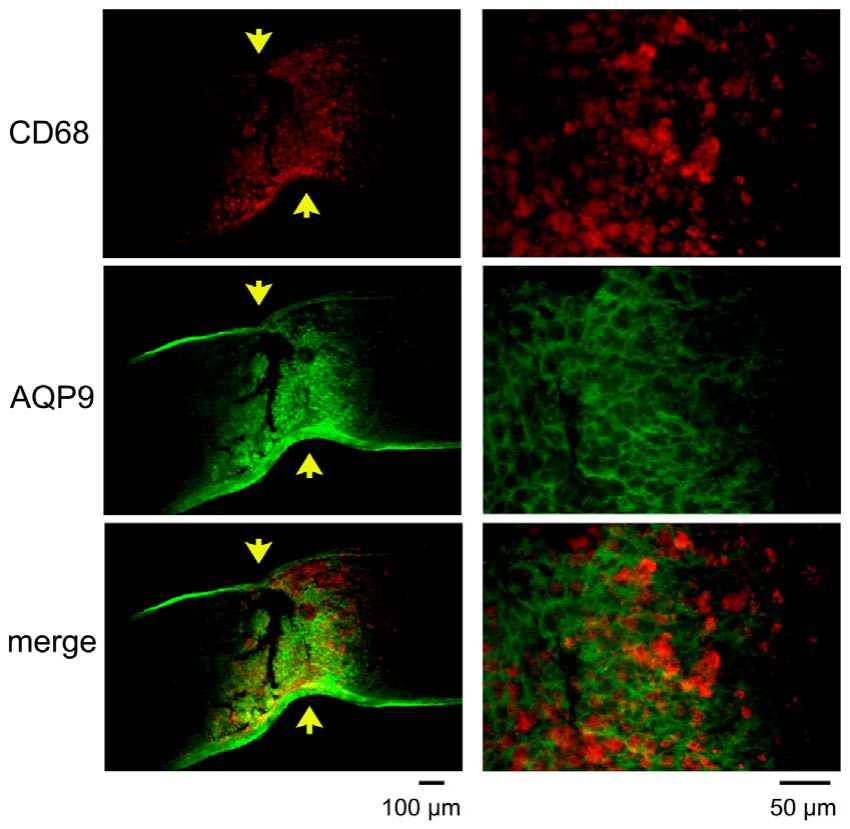
Double labeling of AQP9 and CD68 at the crushed site on day 7. Representative photographs from 3 independent samples are presented with higher magnifications in the right column. Arrows indicate crush sites. CD68 positive cells are present between the AQP9 positive fibrils suggesting that microglia/macrophages are not the cellular sources for AQP9 after crushing the optic nerve.

The expression of AQP9 was enhanced at the crushed site (Figure 6), and the immunoreactivity to AQP9 had a honeycomb appearance. The site was also immunopositive to GFAP (Figure 6A) and nestin (Figure 6B), another intermediate filament protein of astrocytes. There was an accumulation of microglia/macrophages at the crushed site on day 7 as reported earlier (Figure 7). However, immunoreactivity to AQP9 was not co-localized with that of CD68 (Figure 7). Thus, AQP9 was over-expressed in the astrocytes at the crushed site where the expression of AQP4 was down-regulated.

### Blood-optic nerve barrier function

**Figure 8:**
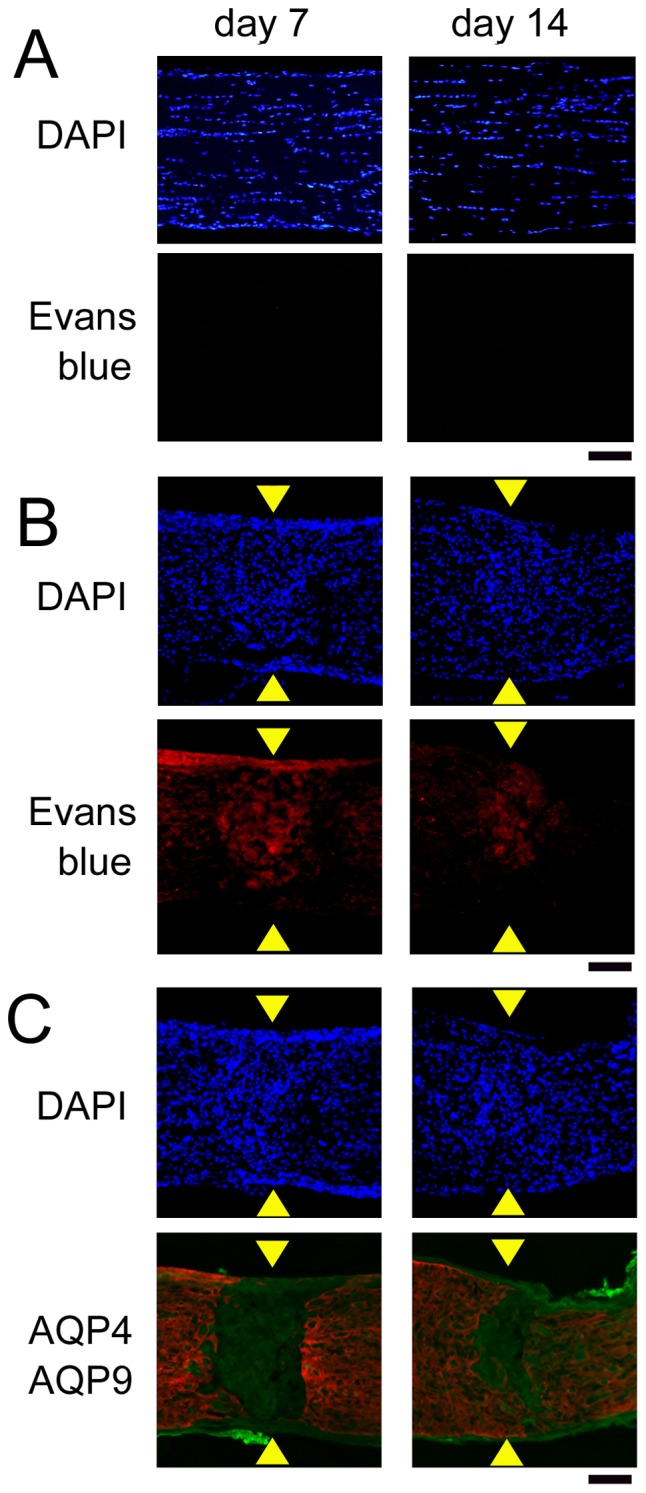
Barrier function determined by extravasated Evans blue. Evans blue dye has not leaked into the optic nerve tissue in the sham controls (A), but it has leaked at the crushed site by the presence of red fluorescence (B). Merged images of AQP4 (red) and AQP9 (green) indicate that extravasated Evans blue was restricted to the crushed site, where AQP4 was negative but AQP9 was expressed (C). The left and right columns are images on days 7 and 14, respectively. Arrowheads indicate crush sites. Bar  = 100 µm.

Representative photomicrographs of extravasated Evans blue dye at the crushed sites on days 7 and 14 are shown in Figure 8. Sections for double labeling with AQP4 and AQP9 were prepared from the same optic nerves, and expression of these proteins is also shown in Figure 8. The red fluorescence of Evans blue dye was observed at the crushed sites (Figure 8B), while such a change was not detected in the sham controls (Figure 8A). The area with red fluorescence appeared to be more restricted on day 14 than on day 7. In addition, these areas with red fluorescence coincided with the areas with AQP9-positive but AQP4-negative astrocytes were present (Figures 8B and 8C).

## Discussion

Our results showed that the temporal and spatial changes in the expression of AQP4 and AQP9 after crushing the optic nerve of rats were quite different. The expression of AQP4 was already increased on day 1 but the level decreased thereafter. The level of AQP4 was significantly lower than that at baseline on days 4 and 7. Immunohistochemistry showed a decrease in the expression of AQP4 and GFAP at the crushed site where AQP4-negative astrocytes were present.

In contrast, AQP9 increased more slowly and reached a peak on day 14. AQP9 was over-expressed at the crushed site where AQP4 was under-expressed.

During cytotoxic edema in the brain, AQP4 deletion decreases the degree of edema [13], and an AQP4 deficiency causes brain swelling in vasogenic edema caused by a blood-brain barrier breakdown [14]. Significant increases of the water content and maximum reduction in the levels of AQP4 on day 7 supports the idea that the down-regulation of AQP4 was closely associated with the crush-induced optic nerve edema. These findings also suggest that crushing the optic nerve causes mainly vasogenic edema due to a disruption of the blood-optic nerve barrier function.

The optic nerve astrocytes supply energy to the axons in the forms of lactate under normal conditions [10], and AQP9 plays a role in the lactate-mediated energy supply [10]. This mechanism may be more important for the axons to survive under hypoxic conditions where oxidative phosphorylation is limited [29].

Traumatic injuries are often accompanied by insufficient blood flow which may impair water and ionic homeostasis, extracellular osmotic pressure, and oxidative phosphorylation. In addition, brain injury causes lactic acidosis [30]. An increase in the level of AQP9 is known to occur in a cultured retinal cell line under hypoxic stress [31], and in the sensory retinas after optic nerve crushing [32], experimental glaucoma [33], and retinal ischemia [22]. There is also evidence that AQP9 is essential for the survival of retinal neurons under oxidative stress [34]. Thus, the gradual increase and spatial distribution of AQP9 expression after optic nerve crushing may counteract the metabolic damage.

We have shown that CD68-positive cells, i.e., microglia/macrophages, were accumulated at the crushed site [28], [35]. These cells were immunopositive for ET-1 and caused a reciprocal activation of astrocytes surrounding the lesion [28]. Along with these changes, remodeling of astrocytes takes place at the crush site [36]. Thus, the properties of the astrocytes at the crushed site may be different from those at the margins of the crush site.

Sun et al. have shown structural change of astrocytes after crushing the optic nerve [36]. In the earlier stage, the optic nerve astrocytes at the crushed site retract their primary processes and simplify their shapes. Despite the hypertrophy of their processes and cell bodies, their spatial coverage is drastically reduced, and their end- feet lose connection to the pia and blood vessels. After 7days post injury, some processes start to re-extend, and their morphological appearance is almost normal by day 14, when some processes restore their contact with the pia and blood vessels [36]. These morphological changes and remodeling of astrocytes may be closely associated with our findings of the changes of AQPs. For example, a loss of contact with blood vessels may cause a reduction of AQP4 expression because AQP4 is most abundant at the astrocytic end-feet [3]. Thus, changes of AQPs would most likely occur where astrocytes undergoes remodeling after the optic nerve is crushed. The expression of AQPs in individual astrocytes may change at different time points after injury. Also, the pattern of expression may change in different processes of the same cells.

The barrier function of the vessels at the crushed site was impaired mainly in the areas where AQP9 was over-expressed and AQP4-negative astrocytes were present. Although the findings are still somewhat contradictory, AQP4 is closely associated with the barrier function [8], [37]. One possibility is that a remodeling by the astrocytes was not complete in these areas. Our data suggest that AQP9 may not fully replace the functions of AQP4 for vascular integrity.

There are limitations to this study. One limitation is that we did not determine how our findings were related to pathological events including neuroinflammation and astrogliosis. In addition, we did not determine whether AQP4 and AQP9 were expressed on the optic nerve vessels by immunohistochemistry. It would be of great importance to determine whether AQP4 inhibition just after the injury is neuro-protective for retinal ganglion cells from optic nerve injuries. This is because AQP4 inhibition is neuroprotective of retinal ischemia [21], but it can exacerbate diabetic retinopathy [38] and light-induced retinal damage [39]. These issues need to be determined in more detail.

In conclusion, the expressions of AQP4 and AQP9 are markedly changed after the optic nerve is crushed in rats. The AQP4-positive astrocytes are replaced by AQP4-negative astrocytes. In addition, AQP9 is over-expressed in astrocytes after the optic nerve is crushed. These changes may compensate for the compromised metabolic conditions but may not fully integrate into the vascular architecture.
